# Long-term remission of acromegaly after somatostatin analogues withdrawal: a single-centre experience

**DOI:** 10.1007/s40618-021-01562-z

**Published:** 2021-05-20

**Authors:** E. Sala, G. Carosi, G. Del Sindaco, R. Mungari, A. Cremaschi, A. L. Serban, C. L. Ronchi, E. Ferrante, M. Arosio, G. Mantovani

**Affiliations:** 1 Endocrinology Unit, Fondazione IRCCS Ca’ Granda, Ospedale Maggiore Policlinico, Via F. Sforza 35, 20122 Milan, Italy; 2 Department of Experimental Medicine, Sapienza University of Rome, Rome, Italy; 3 Department of Clinical Sciences and Community Health, University of Milan, Milan, Italy; 4 Institute of Metabolism and System Research, University of Birmingham, Birmingham, UK

**Keywords:** Acromegaly, Somatostatin analogues, GH, IGF-1, Remission

## Abstract

**Purpose:**

A long-lasting remission of acromegaly after somatostatin analogues (SAs) withdrawal has been described in some series. Our aim was to update the disease evolution after SAs withdrawal in a cohort of acromegalic patients.

**Methods:**

We retrospectively evaluated 21 acromegalic patients previously included in a multicentre study (Ronchi et al. 2008), updating data at the last follow-up. We added further 8 patients selected for SAs withdrawal between 2008–2018. Pituitary irradiation represented an exclusion criterion. The withdrawal was suggested after at least 9 months of clinical and hormonal disease control. Clinical and biochemical data prior and after SAs withdrawal were analysed.

**Results:**

In the whole cohort (29 patients) mean age was 50 ± 14.9 years and 72.4% were females. In 69% pituitary surgery was previously performed. Overall, the median time of treatment before SAs withdrawal was 53 months (IQR = 24–84). At the last follow up in 2019, 23/29 patients (79.3%) had a disease relapse after a median time of 6 months (interquartile range or IQR = 3–12) from the drug suspension, while 6/29 (20.7%) were still on remission after 120 months (IQR = 66–150). IGF-1 levels were significantly lower before withdrawal in patients with persistent remission compared to relapsing ones (IGF-1 SDS: -1.5 ± 0.6 vs -0.11 ± 1, *p* = 0.01). We did not observe any other difference between patients with and without relapse, including SAs formulation, dosage and treatment duration.

**Conclusion:**

A successful withdrawal of SAs is possible in a subset of well-controlled acromegalic patients and it challenges the concept that medical therapy is a lifelong requirement.

## Introduction

Acromegaly is a rare disease caused by chronic growth hormone (GH) and insulin-like growth factor-I (IGF-1) hyper-secretion, associated with increased morbidity and mortality [1–3]. The Endocrine Society Guidelines suggested surgery as primary treatment to normalize GH and IGF-1 secretion[4]. When surgery does not achieve remission, or if it is ineffective or contraindicated, therapeutic options for acromegaly are radiotherapy and/or medical treatment with long-acting somatostatin analogues (SAs) or with GH receptor antagonist (PEG).

First generation SAs, such as octreotide long-acting release (OC) or lanreotide (LR) are considered the first line medical therapy in the treatment of acromegaly [5, 6].

The mechanisms of action of SAs in acromegaly include the inhibition of GH secretion and, to a lesser extent, direct inhibition of IGF-1 secretion [7, 8]. Different studies showed that, in vitro, SAs have also antiproliferative, antiangiogenic and apoptotic effects on pituitary tumours [9, 10].

Many studies also demonstrated that both primary and secondary prolonged treatments with SAs are able to induce an enduring GH/IGF-1 reduction and a pituitary tumour volume decrease in most patients with acromegaly [11, 12]. Nevertheless, they present some side effects, including a possible impairment of glucose tolerance, and apparently the need to be indefinitely continued [13, 14].

Some previous papers demonstrated that a sustained clinical inactivity and stabilization of GH/IGF-1 levels is possible in patients with acromegaly, even after the discontinuation of SAs treatment. However, most of them were isolated case reports or they included only a small number of selected patients with a “transient” disease remission after a relatively short period of drug removal (mostly 6–24 months) [15–19]. Moreover, our group previously observed, in a multicentre study, the possibility of a successful withdrawal of SAs in 5 of 27 good responders after a median period of 24 months [20]. However, to date, it is still uncertain whether SAs may provoke a long-lasting disease remission in GH-secreting tumours after drug discontinuation, similarly to the definitive cure frequently induced by dopamine agonists in prolactinomas. It is still also uncertain whether withdrawal of SAs therapy should be routinely proposed at least to highly responsive patients, thus usefully reducing side effects and costs of a life-lasting pharmacological therapy.

The aim of the present study was to update the results of our previous study [20] trying to establish the basis of a trial of SAs withdrawal in clinical practice. With this aim we examined the evolution of clinical parameters, GH/IGF-1 secretion and tumour mass after a very long-term drug discontinuation in a series of selected patients with acromegaly characterized by an optimal disease control during chronic treatment with different long-acting SAs.

## Materials and methods

### Patients

We retrospectively analysed data at the last available follow up of 21 patients included in a previous multicentre study who regularly refer to our Centre [20]. Moreover, we extended the analysis to 8 acromegalic patients referred to the Endocrinology Unit, Fondazione IRCCS Ospedale Maggiore Policlinico and selected for SAs withdrawal between 2008 and 2018.

As inclusion criteria, all patients had a diagnosis of acromegaly and were treated with long-acting SAs (OC and LR) for a period of at least 12 consecutive months. We included both patients “de novo” and patients treated after surgery.

All patients who previously underwent neurosurgery were evaluated 2 months after surgical treatment for fasting serum GH and post-glucose GH along with IGF-1 concentrations before introducing SAs.

Inclusion criteria were slightly different concerning SAs withdrawal in the previous study and in the new group of patients of the extended analysis. In the first group, patients had to be considered optimally controlled by SAs therapy, as indicated by absence of acromegaly-related signs/symptoms, “safe” GH levels (mean of at least three samples during saline infusion < 2.5 ng/ml) and IGF-1 levels in the lower normal range [21] at a stable dosage of SAs for at least nine consecutive months with a low-medium dosage of SAs. In particular, octreotide was considered low dosage at 10 mg every 28 days, and medium dosage at 20 mg every 28 days, while lanreotide was considered low dosage at 60 mg at least every 28 days or 120 mg at least every 42 days.

In the second and more recent group, inclusion criteria considered the availability of more specific and sensitive assays for GH. In this group, before SAs withdrawal, patients were considered as optimally controlled by therapy, as indicated by the absence of acromegaly-related signs/symptoms, “safe” GH levels (mean of at least three samples during saline infusion < 1 ng/ml) and IGF-1 levels in the lower part of the normal range [22] at a stable dosage of SAs for at least 18 consecutive months with a low-medium dosage of SAs.

Finally, at the time of SAs withdrawal, pituitary tumour or residual tumour volume had to be invisible, reduced, disappeared or at least stable at routine magnetic resonance imaging (MRI). Patients that underwent radiotherapy or radiosurgery were excluded from the study.

Baseline hormonal and radiological data were also recorded and investigated to search for parameters predictive for biochemical behavior after SAs withdrawal. Tumors were classified at the time of diagnosis according to the maximum diameter at MRI into microadenomas (< 10 mm) or macroadenomas (≥ 10 mm).

The Local Ethical Committee (Fondazione IRCCS Ca’ Granda, Milan) approved the protocol study and patients gave their informed written consent to participate.

### Assays

GH was evaluated by IFMA (AutoDelfia, Wallac OY, Turku, Finland) and serum IGF-I levels by RIA Mediagnost, Tubingen, Germany), before 2008. After 2008, GH was assayed with a chemiluminescence method (Immulite 2000, Siemens Medical Solutions Diagnostics, Los Angeles, CA, detection limit of 0.01 μg/L). Standards used for calibration were IS 80/505 from 2008 to July 2010 and IS 98/574 from August 2010. IGF-1 levels were measured by a chemiluminescent immunometric assay (Immulite 2000 IGF-1; Siemens Medical Solutions Diagnostics, Los Angeles, CA) and standards used for calibration were IRR 87/518 from 2008 to April 2017 and IS 02/254 from May 2017.

IGF-1 values were compared with an appropriate age-adjusted range, as reported previously and expressed as SDS. We obtain SDS value accordingly to the methods provided by Chanson and colleagues [23].

### Statistical analysis

Data with normal distribution and non-Gaussian data are expressed as mean ± standard deviation (SD) and as median with interquartile range (IQR), respectively.. GH nadir was defined as the lowest value at any time after 2 h oral glucose tolerance test (OGTT).

A paired Student’s *t* test was performed to compare different variables in case of normally distributed data; non-Gaussian variables were compared using a non-parametric test. Associations between categorical variables were assessed by Fisher’s exact test. Data were analyzed using GraphPad Prism (version 5.0, La Jolla, CA, USA) or SPSS (PASW Version 19.0, SPSS Inc., Chicago, IL, USA). Values of *P* < 0.05 were considered as statistically significant.

## Results

### Patients’ characteristics

We retrospectively analysed data of 29 acromegalic patients, mean age 50 ± 14.9 years, 21 females (72.4%) and 8 males (27.6%); 21 (72.4%) of them were previously reported in the study of Ronchi et al.[20] (group 1), while 8 (27.6%) patients were selected for SAs withdrawal between 2008 and 2018 (group 2).

At diagnosis, MRI revealed the presence of a macroadenoma in 19 (65.5%) and a microadenoma in 8 (27.6%) patients. In one patient, MRI did not show a clear sign of adenoma but only indirect signs of pituitary lesion (stalk deviation and sellar enlargement) and one additional patient refused MRI. In this last patient a computerized tomography (CT) scan did not showed the presence of visible pituitary lesions.

As first line treatment, 20 (69%) patients underwent surgery, in the remaining 9 (31%), surgery was refused or contraindicated. Eleven (37.9%) patients showed a secondary empty sella at the MRI after surgery.

All patients were treated with SAs, in particular, 13 (44.8%) patients were treated with long acting release octreotide (OC) and 16 (55.1%) with slow release lanreotide (LR). Patients were treated for a median time of 53 months (IQR 24–84 months). Mean GH value before starting SAs therapy was 6.04 ± 5.3 ng/ml with a mean GH nadir value after OGTT of 4.2 ± 4.8 ng/ml. IGF-1 values were 6 ± 4.6 SDS.

In all these patients a trial of drug withdrawal was attempted because of optimal disease control. At the last follow up, just before SAs withdrawal, mean GH value was 0.95 ± 0.55 ng/ml (1.03 ± 0.64 ng/ml in group 1 and 0.76 ± 0.22 in group 2) and IGF-1 levels -0.37 ± 1.1 SDS (*p* value < 0.001 compared to IGF-1 before therapy). As a good clinical practice, we a complete liver function was performed panel during SA treatment, and no patient had a concomitant liver failure.

Clinical, hormonal and neuroradiological features of all treated patients are shown in Table 1.

**Table 1:** Clinical, neuroradiological and hormonal characteristics of all patients, collected before starting somatostatin analogues

Characteristics	Values
Included in the previous study (group 1), *n* (%)	21 (72)
New patients (group 2), *n* (%)	8 (28)
Age, years	50 ± 14.9
Female, *n* (%)	21 (72)
**Neuroradiological findings at diagnosis**	
Macroadenoma, *n* (%)	19 (66)
Microadenoma, *n* (%)	8 (28)
Indirect signs of adenoma, *n* (%)	1 (3)
MRI not performed, *n* (%)	1 (3)
**TNS before starting SAs**	
Yes	20 (69)
No	9 (31)
**Hormonal findings before starting SAs**	
IGF-1, ng/ml (SDS)	548 ± 302 (6 ± 4.6)
Mean GH, ng/ml	6.04 ± 5.3
GH nadir, ng/ml	4.2 ± 4.8

Data are expressed as number, percentage (%) and mean ± SDS

*MRI* magnetic resonance imaging, *SAs* somatostatin analogues ,*TNS* transphenoidal surgery

### Clinical and biochemical behaviour after SAs withdrawal

At the last available follow up in 2019, 6/29 patients (20.7%), even after SAs withdrawal, persistently showed a good disease control (long term remission group). The other 23/29 patients (79.3%) had a disease relapse after drug withdrawal and, at the last visit, 19 were still in medical therapy for active acromegaly. In particular, 1 patient was on dopamine agonist (DA), 6 on LR, 1 on LR and DA, 8 on OC, 1 on pasireotide (PAS) and 2 patients on pegvisomant (PEG). Two patients were successfully treated with gamma-knife radiosurgery and the remaining two underwent a second TNS surgical approach with disease remission.

### Relationship between long-term remission and clinical parameters

The group of 6 patients well controlled after long term SAs withdrawal showed, at the last follow-up, IGF-1 levels of 165 ± 39.4 ng/ml with SDS + 0.3 ± 1.6, with a mean of GH levels of 1.4 ± 0.7 ng/ml. The median follow up time in these patients was 120 months (IQR 66–150). OGTT was performed at least once in 5/6 patients and GH nadir was 0.41 ± 0.18 ng/ml at the last available test after 57.6 ± 30 months after SAs withdrawal. In this group, 4/6 patients were already in remission at the end of the previous study of Ronchi et al. (updated median follow up time of 132 months, IQR 111–153). At the end of that previous study, 5 (18.5%) patients resulted in remission after a median follow up time of 24 months (IQR 21–36 months) but 1 patient had a relapse 30 months after withdrawal so that therapy with SAs (OC) was re-initiated. Pituitary imaging remained stable after drug withdrawal in all these 6 patients.

A comparison of clinical, neuroradiological and hormonal features between patients with and without relapse is shown in Table 2. Comparing patients in persistent remission with patients relapsed after drug withdrawal, we did not find any difference in radiological or therapeutic characteristics, see Table 2. Nevertheless, at the last follow up before withdrawal, our data showed IGF-1 levels significantly lower in patients with persistent remission compared to patients with subsequently relapse (IGF-1 SDS − 1.5 ± 0.6 vs − 0.11 ± 1 with *p* = 0.01 see Table 2). In particular, 4 out of 6 (66.6%) patients had IGF1 levels under the lower limit of normality before withdrawal, while 2 patients (33.3%) had IGF-1 in the lower normal range. On the contrary, no differences in the last GH levels before withdrawal were found (0.96 ± 0.6 vs 0.96 ± 0.45 ng/ml in patients with relapse and persistent remission, respectively; *p* value 1.0, see Table 2).

**Table 2:** Clinical, hormonal and neuroradiological evaluations in the long-term remission group compared to the group with disease relapse

	Long term remission	Disease relapse	*p* value
Number of patients, *n*	6	23	NA
Age, years	48 ± 17	51 ± 14	0.7
Sex (M/F)	1/5	7/16	0.6
Macro, *n* (%) Micro, *n* (%) No adenoma, *n* (%)	5 (83) 1 (17) 0	14 (61) 7 (30) 2 (9)	0.6 0.6 1.0
TNS before SAs, *n* (%)	5 (83)	15 (65)	0.6
Hypopituitarism, *n* (%)	2 (40)	8 (33)	1.0
Duration of therapy, months	64.6 ± 49	60.1 ± 38.8	1.0
**Hormonal data before starting SAs**			
IGF-1, SDS	5.4 ± 2.3	6.2 ± 5	0.7
Mean GH, ng/ml	5.1 ± 3.3	6.3 ± 5.8	0.6
GH nadir, ng/ml	4.9 ± 5.6	5.7 ± 5.1	0.7
**Hormonal data before SAs withdrawal**			
IGF-1, SDS	−1.5 ± 0.6	−0.11 ± 1	0.01*
Mean GH, ng/ml	0.96 ± 0.45	0.96 ± 0.61	1.0

Data are expressed as number, percentage (%) and mean ± SDS; p-value is considered statistically significant if < 0.05

*NA* not applicable, *Macro* macroadenoma, *Micro* microadenoma, *TNS* transphenoidal surgery, *SAs* somatostatin analogues

**Fig. 1:**
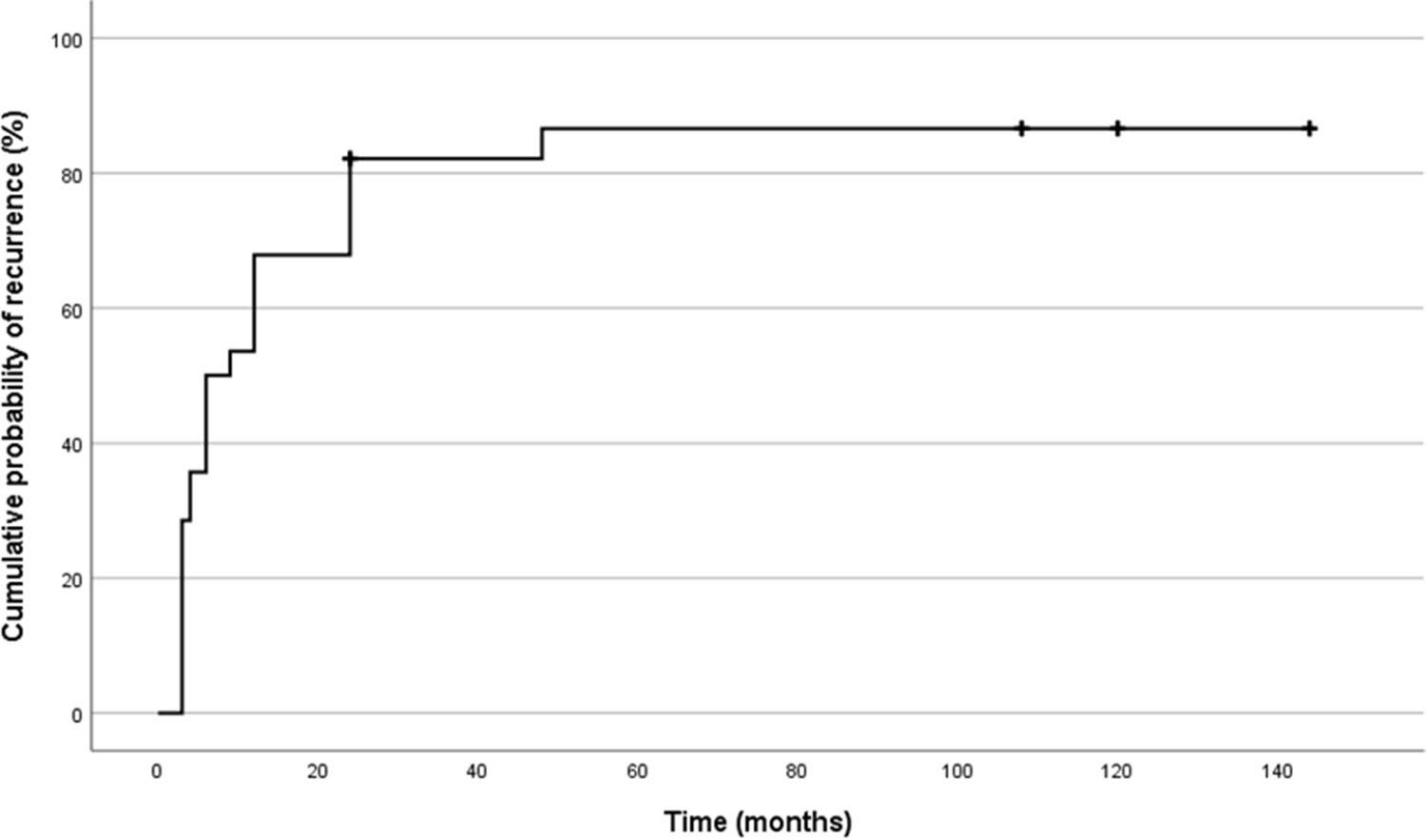
Kaplan–Meier curve showing cumulative probability of disease recurrence after somatostatin analogues withdrawal. We found a higher possibility of recurrence in the first months and no recurrence was encountered after 48 months of drug suspension.

Concerning the timing of relapse, our data showed a median time of relapse of 6 months (IQR 3–12), and we specifically counted 8 patients relapsing in the first 3 months after withdrawal (35%), 5 in 6 months (22%), 5 in 12 months (22%), 4 in 24 months (17%) and the remaining one in 48 months (4%) (see Fig. 1). Interestingly, even with a long follow up (up to 156 months), no patients relapsed after more than 48 months, see Fig. 1.

### Multiple withdrawal

In a small subgroup of 5 patients (17.2%), 3 males (60%), mean age 44 ± 22, withdrawal was tried at least twice during the follow up. The data on the first withdrawal in these patients were previously described in the previous study on this topic [20]. Three (60%) of them at diagnosis had a macroadenoma, 1 (20%) patient had a microadenoma, while the last patient refused MRI. The 3 patients with a macroadenoma underwent TNS.

Three (60%) patients were treated with OC and 2 (20%) with LR. After a median period of 72 months (IQR 36–97), was tried the first withdrawal. During SAs therapy, at last follow up just before the first withdrawal, mean GH value was 0.8 ± 0.5 ng/ml and IGF-1 159.9 ± 45.1 ng/ml, SDS − 1.4 ± 2.4. No differences between these patients and the others with only one withdrawal were present.

All patients had a recurrence after a median period of 4 months (IQR 3–18) after the withdrawal and the previous therapy was restarted.

After a median period of 108 months (IQR 36–162), SAs therapy was again stopped. At that time mean GH was 0.74 ± 0.59 ng/ml and IGF-1 119.4 ± 33.1 ng/ml with SDS -0.1 ± 0.8. After a median period of 24 months (IQR 6–31), all the patients had a second relapse starting again the same therapy and achieving immediately a good control. At the last follow up, 4 patients are still in good control of therapy with SAs. In one patient, after 60 months of optimal control, withdrawal was tried again. At the last follow up, after 24 months from the suspension he is still in good control with IGF-1 in the normal adjusted range for age and sex and normal GH level.

## Discussion

These data confirm, as demonstrated in other previous studies on this topic [15–19], that chronic treatment with long-acting SAs may be able to induce a long-term disease remission in a subgroup of patients with acromegaly. In fact, our data affirm with a similar percentage the presence of a persistent remission at long term follow up, with persistence of constantly ‘safe’ GH levels, normal IGF-1 concentrations, and stable neuroradiological imaging. Moreover, the present study adds an interesting clue on the timing of relapse. In fact, according with other studies published in literature, we found that all patients had a relapse between 3 and 48 months after SAs withdrawal [15–19]. After a period of 48 months, even prolonging our follow up to 156 months, no one showed a relapse.

The main aim of this study was to establish the rationale for a periodic SAs withdrawal in clinical practice. Our data interestingly showed that in the group of patients with persistent remission, IGF-1 levels at the last follow up before withdrawal were significantly lower compared to the IGF-1 levels in the group with relapse. On the contrary, no differences in GH levels or other clinical, radiological or therapeutic parameters were found between the two groups (see Table 2). If confirmed in larger prospective studies, this data could be interesting to clarify, in clinical practice, when SAs withdrawal is possible and hopefully long lasting.

Our results are important to expand and clarify the different and controversial results in the literature, in most cases limited by the small number of patients included [15, 17]. Moreover, some data came from isolated case reports [18]. In all these studies, no clear predictive factors for long-term remission were identified, except for the study of Hatipoglu and colleagues [17]. The authors suggested that in a small cohort of 16 patients, GH levels before SAs withdrawal are lower in patients with persistent remission.

The study with the largest cohort on this topic (58 patients) showed, differently from our data, that long-term remission after OC discontinuation was possible only in 4 patients after 60 week follow up [19]. The authors, therefore, suggested that a long-term remission after SAs withdrawal is an uncommon and frequently unsustainable event, not supporting the recommendation of a systematic withdrawal in controlled patients.

On the contrary, a common finding in all published studies, similarly to our data, is the timing of relapsing. In fact, this event is more common in the first 3–6 months after withdrawal (median of 6 months with an IQR of 3–12). Interestingly, with a prolonged follow up (up to 156 months in our study), we did not observe any relapse after 48 months of follow up. This is important to suggest clinicians the best follow up in acromegalic patients after medical therapy withdrawal, even if our series is still too small to draw definitive conclusions.

In summary, consistent with our data, SAs therapy seems to be able to induce a long-term remission at least in a subgroup of patients with acromegaly (21%), challenging the previously held concept that medical therapy is always a lifelong requirement. Our data suggest the possibility for a SAs discontinuation especially in those patients achieving an optimum IGF-1 control with low dosage of SAs.

Moreover, in view of programming SAs discontinuation and scheduling follow-up intervals, our data confirm that recurrence is more common within 3–6 months from drug suspension and unlikely after 48 months. According to this observation, we can suggest a close follow-up with a dedicated endocrinologist after drug discontinuation in the first 48 months (e.g., every 3–6 months in the first year, every 6 months in the second and then, after 48 months from the discontinuation, annually).

## Limitation of the study

We acknowledge that this study is limited by the retrospective nature of the analysis. The relatively small number of subjects included and the length of follow up in some patients limit the conclusions and preclude the identification of other potential prognostic factors. The suggestion that, in a small subgroups of patients, SAs may be able to induce long term remission of acromegaly, needs to be confirmed in larger prospective studies. For example, a prospective study with a non-treated control groups would be important to detect the impact of the SAs vs the natural history of GH secreting pituitary adenoma. Nevertheless, our findings are potentially important to decrease sanitary costs of health services, medical therapies in chronic patients and to reduce drug related side effects.

## Data Availability

The datasets used and analysed during the current study are available from the corresponding author on reasonable request.
